# Tricomponent Exciplex Emitter Realizing over 20% External Quantum Efficiency in Organic Light‐Emitting Diode with Multiple Reverse Intersystem Crossing Channels

**DOI:** 10.1002/advs.201801938

**Published:** 2019-05-15

**Authors:** Ming Zhang, Wei Liu, Cai‐Jun Zheng, Kai Wang, Yi‐Zhong Shi, Xing Li, Hui Lin, Si‐Lu Tao, Xiao‐Hong Zhang

**Affiliations:** ^1^ School of Optoelectronic Science and Engineering University of Electronic Science and Technology of China (UESTC) Chengdu 610054 P. R. China; ^2^ Institute of Functional Nano & Soft Materials (FUNSOM) Soochow University Suzhou 215123 P. R. China

**Keywords:** exciplex, organic light‐emitting diodes (OLEDs), reverse intersystem crossing channel, thermally activated delayed fluorescence, tricomponent

## Abstract

With the naturally separated frontier molecular orbitals, exciplexes are capable of thermally activated delayed fluorescence emitters for organic light‐emitting diodes (OLEDs). And, the current key issue for exciplex emitters is improving their exciton utilization. In this work, a strategy of building exciplex emitters with three components is proposed to realize multiple reverse intersystem crossing (RISC) channels, improving their exciton utilization by enhancing upconversion of nonradiative triplet excitons. Accordingly, a tricomponent exciplex DBT‐SADF:PO‐T2T:CDBP is constructed with three RISC channels respectively on DBT‐SADF, DBT‐SADF:PO‐T2T, and CDBP:PO‐T2T. Furthermore, its photoluminescence quantum yield and rate constant of the RISC process are successfully improved. In the OLED, DBT‐SADF:PO‐T2T:CDBP exhibits a remarkably high maximum external quantum efficiency (EQE) of 20.5%, which is the first report with an EQE over 20% for the OLEDs based on exciplex emitters to the best of our knowledge. This work not only demonstrates that introducing multiple RISC channels can effectively improve the exciton utilization of exciplex emitters, but also proves the superiority of the tricomponent exciplex strategy for further development of exciplex emitters.

## 1 Introduction

Exciplexes arouse enormous attention due to their unique optoelectronic characteristics and great applications in organic light emitting diodes (OLEDs).^1^, ^2^, ^3^, ^4^, ^5^, ^6^, ^7^, ^8^, ^9^, ^10^, ^11^, ^12^, ^13^, ^14^, ^15^, ^16^ Formed between electron‐donating (D) and electron‐accepting (A) molecules, all exciplexes possess intermolecular charge‐transfer (CT) transition and locate their highest occupied molecular orbital (HOMO) and lowest unoccupied molecular orbital (LUMO) on the D and A molecules, respectively. With these completely separated frontier molecular orbitals, exciplexes naturally have extremely small energy gaps (Δ*E*_ST_s) between their lowest singlet and triplet (S_1_ and T_1_) energy levels.^1^, ^13^, ^14^ According to our previous work, by further controlling the T_1_ energy levels of two constituting molecules higher than that of exciplex, exciplexes can exhibit significant thermally activated delayed fluorescence (TADF) behaviors,^12^, ^13^, ^14^ which will promote the utilization of electrogenerated triplet excitons in the OLEDs. Besides, as the constituting D and A materials are generally capable of hole‐ and electron‐transporting properties, respectively, exciplexes would possess bipolar transporting properties, and even reach a charge‐transporting balance by adjusting the mixing ratio between D and A, which would be helpful to further simplify the structures and lower the driving voltages for OLEDs.^2^, ^7^, ^14^, ^17^ Therefore, exciplexes were widely investigated as the TADF emitters or hosts of OLEDs in recent years.^1^, ^2^, ^3^, ^5^, ^6^, ^7^, ^8^, ^9^, ^10^, ^11^, ^12^, ^13^, ^14^, ^17^, ^18^, ^19^, ^20^, ^21^, ^22^, ^23^, ^24^, ^25^, ^26^, ^27^, ^28^, ^29^, ^30^, ^31^, ^32^, ^33^, ^34^, ^35^

As the exciplexes can realize the balanced charge transport and suppress the triplet–triplet annihilation by upconvert triplet excitons to singlet excitons, the OLEDs using exciplexes as the hosts have realized remarkable progress till now. The traditional fluorescent emitters, phosphorescent emitters, and TADF emitters all realized superior performance in exciplex hosts than in conventional host materials, exhibiting lower driving voltages and improved efficiency roll‐off in the devices.^17^, ^26^, ^29^, ^36^, ^37^, ^38^, ^39^ However, by using exciplexes as the TADF emitters in the OLEDs, only limited progress was realized. In 2012, Goushi et al. reported an OLED with a maximum external quantum efficiency (EQE) of 5.4% by using m‐MTDATA:3TPYMB exciplex as the emitter, demonstrating the TADF characteristic of exciplexes for the first time.^1^ In 2013, Hung et al. reported an interface exciplex‐based OLED with a maximum EQE of 7.7%, suggesting the superiority of exciplex emitters on device design.^18^ In 2014, Li et al. reported OLEDs based on mCP:HAP‐3MF emitter with a maximum EQE of 11.3%.^21^ In 2015, our group reported an exciplex‐based TADF‐OLEDs by employing a bipolar constituting molecule, and achieved a maximum EQE of 15.4%.^13^ Although these reports successfully demonstrate the OLEDs using exciplexes as the emitters have the potential to realize full excitons utilization by harvesting nonradiative triplet excitons via the TADF channel, their efficiencies are far from the theoretical limit (assuming the light out‐coupling efficiency is 20–30%). Developing strategies for further improving the exciton utilization of exciplex emitters is highly desired.

**Figure 1:**
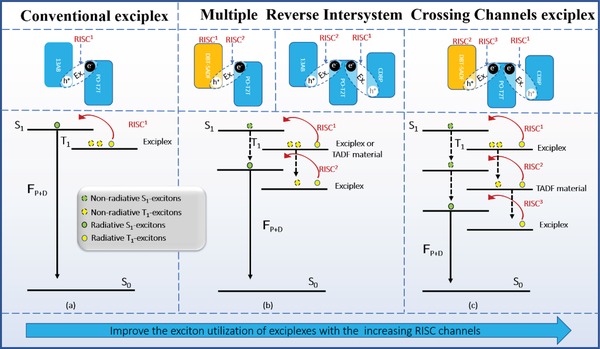
Energy transfer diagrams of the conventional bicomponent TADF exciplexes, TADF‐assistant bicomponent TADF exciplexes, and novel tricomponent TADF exciplexes (blue: conventional constituent material, orange: single‐molecule TADF emitter). S_1_, T_1_, and S_0_ are the singlet excited state, triplet excited state, and ground state, respectively. RISC is reverse intersystem crossing from nonradiative T_1_ to radiative S_1_. F_P+D_ stands for prompt and delayed fluorescence.

It is generally understood that the exciton utilization of TADF emitter is decided by its reverse intersystem crossing efficiency of triplet excitons (*Φ*_RISC_) and fluorescence quantum yield of singlet excitons (*Φ*_f_).^1^, ^40^, ^41^, ^42^, ^43^, ^44^, ^45^ For exciplex TADF emitters, as their HOMO and LUMO are respectively located on different constituting molecules, their *Φ*_f_s are hard to be evidently improved due to such natural separation between HOMO and LUMO. As a result, further enhancing *Φ*_RISC_s of exciplex emitters should be the most possible way to improve their excitons utilization. As shown in **Figure** **1**, conventional exciplexes only have one intrinsic reverse intersystem crossing (RISC) channel, which limits the upconversion of triplet excitons. In 2016, our group increased the RISC channels of the exciplexes by introducing a TADF emitter as one constituting molecule, and MAC:PO‐T2T exciplex realized a high EQE of 17.8% in the OLED,^14^ which is the highest efficiency among the reported OLEDs using exciplex as emitters till now. However, there is still conspicuous room for further improving the exciton utilization of exciplex emitters.

To address this issue, in this work, we proposed a novel strategy of building exciplex emitters with three components to realize multiple RISC channels in the exciplex systems, improving their excitons utilization with further enhanced *Φ*_RISC_s. As shown in Figure 1, such tricomponent exciplex emitters should be capable of forming two TADF exciplexes in the systems, and have two intrinsic RISC channels on both exciplexes. The lower‐energy exciplex could be beneficial due to the energy transfer between two exciplexes. Moreover, by further introducing a TADF emitter as a component as shown in Figure 1, the tricomponent exciplex emitters can even possess three RISC channels, thus have the possibility to exhibit superior performance than current reported exciplex emitters. According to our novel strategy, two tricomponent exciplex emitters, 13AB ((1,3‐bis(9,9‐dimethylacridin)) benzene):PO‐T2T ((1,3,5‐triazine‐2,4,6‐triyl) tris(benzene‐3,1‐diyl) tris(diphenylphosphine oxide)):CDBP (4,4′‐bis(9‐carbazolyl)‐2,2′‐dimethylbiphenyl) and DBT‐SADF (2‐(spiro[acridine‐9,9′‐fluoren]‐10‐yl)dibenzo[*b*,*d*]thiophene 5,5‐dioxide):PO‐T2T:CDBP, were constructed. Among them, 13AB, PO‐T2T, and CDBP are conventional constituting materials, while DBT‐SADF is a TADF constituting material; and 13AB, CDBP, and DBT‐SADF all can act as the D molecule to form TADF exciplexes with PO‐T2T. Particularly, CDBP:PO‐T2T has the highest energy gap than 13AB:PO‐T2T and DBT‐SADF:PO‐T2T. Therefore, tricomponent exciplex 13AB:PO‐T2T:CDBP will emit the emission of 13AB:PO‐T2T and possess two RISC channels on 13AB:PO‐T2T and CDBP:PO‐T2T; while DBT‐SADF:PO‐T2T:CDBP will emit the emission of DBT‐SADF:PO‐T2T and even have three RISC channels respectively on DBT‐SADF, DBT‐SADF:PO‐T2T, and CDBP:PO‐T2T. With the additional RISC channel, tricomponent exciplexes successfully exhibit much higher photoluminescence quantum yields (*Φ*_PL_s) and rate constants of RISC process (*k*_RISC_s) than their corresponding bicomponent exciplexes (40.0% and 0.64 × 10^5^ for 13AB:PO‐T2T:CDBP vs 15.0% and 0.05 × 10^5^ for 13AB:PO‐T2T; 61.0% and 14.2 × 10^5^ for DBT‐SADF:PO‐T2T:CDBP vs 38.0% and 1.17 × 10^5^ for DBT‐SADF:PO‐T2T). In the devices, 13AB:PO‐T2T:CDBP exhibits a maximum EQE of 15.5%, while 13AB:PO‐T2T exhibits a maximum EQE of 12.4%. Moreover, with the three RISC channels, DBT‐SADF:PO‐T2T:CDBP exhibits a remarkably high maximum EQE of 20.5% in the device, which is much higher than DBT‐SADF:PO‐T2T with a maximum EQE of 16.9%. To the best of our knowledge, this is the first report with the EQE achieving over 20% at room temperature for the OLEDs based on exciplex emitters.^1^, ^2^, ^4^, ^5^, ^6^, ^7^, ^8^, ^9^, ^10^, ^11^, ^12^, ^13^, ^14^, ^17^, ^18^, ^19^, ^20^, ^21^, ^30^, ^31^, ^46^, ^47^, ^48^ The outstanding performance of the DBT‐SADF:PO‐T2T:CDBP‐based OLEDs not only demonstrates that introducing multiple RISC channels can effectively improve the exciton utilization of exciplex emitters, but also proves the superiority of our novel tricomponent exciplex strategy for further development of exciplex emitters.

## 2 Results and Discussions

**Figure 2:**
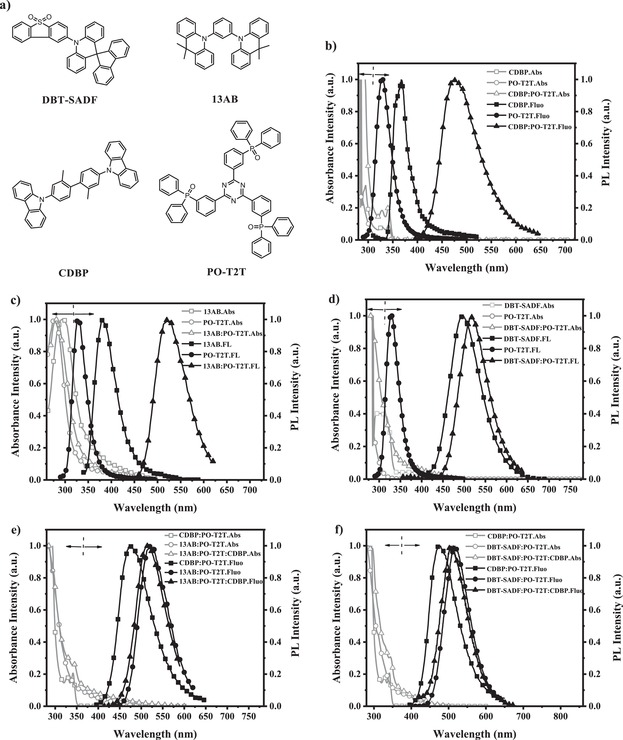
a) Molecular structures of DBT‐SADF, 13AB, CDBP, and PO‐T2T. b) Absorption and PL spectra of the CDBP:PO‐T2T and their pristine materials in thin solid films (PO‐T2T in toluene solution). c) Absorption and PL spectra of the 13AB:PO‐T2T (5:5) and their pristine materials in thin solid films (PO‐T2T in toluene solution). d) Absorption and PL spectra of the DBT‐SADF:PO‐T2T (6:4) and their pristine materials in thin solid films (PO‐T2T in toluene solution). e) Absorption and PL spectra of 13AB:PO‐T2T:CDBP (1:1:1), 13AB:PO‐T2T (5:5), and CDBP:PO‐T2T(5:5) in thin solid films. f) Absorption and PL spectra of DBT‐SADF:PO‐T2T:CDBP (2:5:3), DBT‐SADF:PO‐T2T (6:4), and CDBP:PO‐T2T(5:5) in thin solid films.

Molecular structures of the constituting materials are shown in **Figure** **2**a. Common carrier transporting materials 13AB, CDBP, and PO‐T2T were directly purchased from commercial sources, and DBT‐SADF was newly designed and synthesized. Synthetic route and detailed synthesis of DBT‐SADF are depicted in the Supporting Information. DBT‐SADF was fully characterized and confirmed with ^1^H, ^13^C nuclear magnetic resonance spectroscopies and mass spectroscopy, and purified by sublimation before further characterizations. As shown in Figure S1a in the Supporting Information, the fluorescence and phosphorescence of DBT‐SADF doped in mCP (1,3‐Di‐9‐carbazolybenzene) film were investigated at 77 K, and its Δ*E*_ST_ is determined to be 0.030 eV from the difference between the peaks of the fluorescence and phosphorescence spectra, which is basically required for TADF emitters. To further demonstrate the TADF characteristic of DBT‐SADF, the temperature‐dependent transient photoluminescence (PL) decays of DBT‐SADF doped in mCP film were shown in Figure S1b in the Supporting Information. From 100 to 300 K, the delayed parts are remarkably increased due to the enhanced RISC process with the increased temperature, which is the direct evidence for TADF behavior of DBT‐SADF.^1^, ^40^ Moreover, DBT‐SADF‐based OLEDs were also fabricated by using mCP as the host material. The optimized device structure and the device performance are shown in the Supporting Information. The device exhibits a maximum EQE of 14.0%, which is far higher than the theoretical limit of 5% EQE for conventional fluorescent emitters, further demonstrating the TADF characteristic of DBT‐SADF. To better understand our exciplex systems, we also investigated the fluorescence and phosphorescence of 13AB pure film at 77 K (Figure S2b, Supporting Information), and its Δ*E*_ST_ is determined to be 0.56 eV. The temperature‐dependent transient photoluminescence decays of 13AB were also measured, and the trend of delayed parts is consistent with blank reference at 300 K, which is the direct evidence for non‐TADF behavior of 13AB. Besides, we also investigated the carrier transport characteristics of 13AB and DBT‐SADF, and both the 13AB and DBT‐SADF are hole‐dominated materials.

Figure 2b,d shows absorption and PL spectra of three bicomponent mixtures CDBP:PO‐T2T, 13AB:PO‐T2T, and DBT‐SADF:PO‐T2T and their constituting molecules. The absorption spectra of three bicomponent mixtures are nearly identical to those of the constituting molecules, which suggest that the formation of new ground‐state transitions does not occur in the mixed films. Meanwhile, the PL spectra of the mixtures are significantly red‐shifted relative to those of the constituting molecules. The emissions of CDBP:PO‐T2T, 13AB:PO‐T2T, and DBT‐SADF:PO‐T2T are peaked at 478, 522, and 516 nm, whereas the emission peaks of CDBP, 13AB, PO‐T2T, and DBT‐SADF are at 367, 381, 327, and 497 nm, respectively. These results indicate the formation of new excited states in the mixed films. Besides, the PL spectra of CDBP:PO‐T2T, 13AB:PO‐T2T, and DBT‐SADF:PO‐T2T correspond to the energies of 2.60, 2.38, and 2.41 eV, which match well to the differences between the LUMO energy level of PO‐T2T (−3.22 eV) and the HOMO energy levels of CDBP (−5.83 eV), 13AB (−5.60 eV), and DBT‐SADF (−5.65 eV). (All the energy levels were newly measured in our laboratory and shown in Figure S3 in the Supporting Information.) Particularly, as the emission peak of DBT‐SADF:PO‐T2T is close to that of DBT‐SADF neat film (516 and 514 nm), we further investigated PL spectra and transient fluorescence decays of DBT‐SADF:mCP (1%, 10%, 20%, 5%, 70%, 100% doped) and DBT‐SADF:POT2T (3:7, 4:6, 5:5, 6:4) films with varying concentration of DBT‐SADF, and the results are shown in Figure S4 in the Supporting Information. Increasing the weight ratio of DBT‐SADF from 1% to 100%, the PL spectra of DBT‐SADF:mCP films are evidently changed with the peaks red‐shifted from 490 to 514 nm and the full‐widths at half‐maximum enlarge from 72 to over 100 nm. These results suggest the emission of DBT‐SADF:mCP should be from the intramolecular CT transition of DBT‐SADF, which is sensitive with the environmental polarity increased with more D–A structure DBT‐SADF molecules.^49^ Reversely, all DBT‐SADF:POT2T films exhibit similar PL spectra peaked around 516 nm, suggesting their emission should be from the exciplex between DBT‐SADF and PO‐T2T. Therefore, all three bicomponent mixed films can form exciplexes. Moreover, determined from the highest energy vibronic sub‐band of their phosphorescence spectra at 77 K (Figure S2, Supporting Information), the T_1_ energy levels of 13AB, CDBP, DBT‐SADF, and PO‐T2T are 3.01, 3.02, 2.53, and 2.95 eV, respectively. These values are evidently higher than the energy values of their corresponding exciplexes, which can ensure the exciplexes harvest both singlet and triplet excitons.^12^, ^13^, ^14^ In our previous report, we have demonstrated the TADF characteristic of CDBP:PO‐T2T.^12^ Thereby, we here measured the Δ*E*_ST_s and temperature‐dependent transient PL decays for 13AB:PO‐T2T and DBT‐SADF:PO‐T2T to prove their TADF behaviors. As shown in Figure S5 in the Supporting Information, 13AB:PO‐T2T and DBT‐SADF:PO‐T2T respectively exhibit extremely small Δ*E*_ST_s of 0.047 and 0.032 eV from the differences between the peaks of their fluorescence and phosphorescence spectra at 77 K. And from 100 to 300 K, more significant decays in the microsecond range were observed with increased temperatures, confirming the existence of TADF characteristic. As shown in Figure 1, CDBP:PO‐T2T and 13AB:PO‐T2T are conventional bicomponent TADF exciplexes, and DBT‐SADF:PO‐T2T is the TADF‐assistant bicomponent TADF exciplex.

Based on three bicomponent TADF exciplexes, we constructed two tricomponent exciplexes, 13AB:PO‐T2T:CDBP and DBT‐SADF:PO‐T2T:CDBP, according to our strategy. As shown in Figure 2e,f, both the two tricomponent mixtures exhibit cumulative absorptions compared with the bicomponent exciplexes, suggesting still no formation of new ground‐state transitions. And the PL spectra of 13AB:PO‐T2T:CDBP and DBT‐SADF:PO‐T2T:CDBP are almost consistent with those of 13AB:PO‐T2T and DBT‐SADF:PO‐T2T, respectively. These results do not only indicate that exciplex would be formed in the tricomponent mixtures as well, but also prove complete energy transfer from higher‐energy CDBP:PO‐T2T to lower‐energy 13AB:PO‐T2T or DBT‐SADF:PO‐T2T. We can realize the lower‐energy exciplex emission with the assistance of the higher‐energy exciplex in the tricomponent mixture. Therefore, as shown in Figure S6 in the Supporting Information, 13AB:PO‐T2T:CDBP will possess two RISC channels on 13AB:PO‐T2T and CDBP:PO‐T2T; while DBT‐SADF:PO‐T2T:CDBP can even have three RISC channels on DBT‐SADF, DBT‐SADF:PO‐T2T, and CDBP:PO‐T2T, respectively, which should benefit the upconversion of nonradiative triplet excitons to radiative singlet excitons.

We then measured the *Φ*_PL_s of 13AB:PO‐T2T, 13AB:PO‐T2T:CDBP, DBT‐SADF:PO‐T2T, and DBT‐SADF:PO‐T2T:CDBP by using an integrating sphere under oxygen‐free condition. And the absolute *Φ*_PL_s are measured to be 15.0% for 13AB:PO‐T2T, 40.0% for 13AB:PO‐T2T:CDBP, 38.0% for DBT‐SADF:PO‐T2T, and 61.0% for DBT‐SADF:PO‐T2T:CDBP. With the increasing RISC channels, the *Φ*_PL_s of the exciplexes are evidently enhanced, and the highest *Φ*_PL_ of 61.0% is realized with three RISC channels. These results indicate the multiple RISC channels in tricomponent exciplexes will indeed enhance the utilization of excitons. To better understand the superiority of multiple RISC channels in tricomponent exciplexes, we further measured the transient PL decays of four exciplex emitters at room temperature and calculated their key kinetic parameters. For blue and green fluorescent emitters, the internal conversion process of singlet excitons could be ignored compared with fluorescence decay and intersystem crossing process of singlet excitons,^41^, ^42^, ^43^ thus the calculations were carried out via the following equations^41^, ^42^

(1) $$k_{p}=\frac{1}{\tau_{p}}$$

(2) $$k_{F}=k_{p}\;\Phi_{F}$$

(3) $$k_{\mathrm{ISC}}=k_{P}\;\left(1-\Phi_{F}\right)$$

(4) $$k_{\mathrm{TADF}}=\frac{\Phi_{\mathrm{TADF}}}{\Phi_{\mathrm{ISC}}\tau_{\mathrm{TADF}}}$$

(5) $$k_{\mathrm{RISC}}=\frac{k_{p}k_{d}}{k_{\mathrm{ISC}}}\;\frac{\Phi_{\mathrm{TADF}}}{\Phi_{F}}$$

where *Φ*_F_ and *Φ*_TADF_ are the quantum efficiencies of prompt and delayed fluorescence; *Φ*_ISC_ is the efficiency of ISC process; *k*_p_, *k*_F_, *k*_ISC,_ *k*_TADF_, and *k*_RISC_ are rate constants of prompt fluorescence, fluorescence decay, ISC process from S_1_ to T_1_ state, delayed fluorescence decay, and RISC process, respectively. *Φ*_F_ and *Φ*_TADF_ of these exciplex emitters are estimated from the *Φ*_PL_ with a relative ratio which was calculated from the transient PL results.^41^ Consequently, we got all key kinetic parameters of four exciplex emitters and list them in **Table** **1**. With naturally separated HOMO and LUMO distributions, four exciplexes exhibit relatively small *k*_F_s and relatively large *k*_ISC_s, resulting in all four *Φ*_F_s with small values. However, for the RISC process, *k*_RISC_s are evidently increased from 0.05 × 10^5^ for 13AB:PO‐T2T with only one RISC channel to 0.64 × 10^5^ and 1.17 × 10^5^ for 13AB:PO‐T2T:CDBP and DBT‐SADF:PO‐T2T with two RISC channels, and finally to 14.2 × 10^5^ for DBT‐SADF:PO‐T2T:CDBP with three RISC channels. As a result, *Φ*_TADF_s are also increased from 6.7% for 13AB:PO‐T2T to 28.0% for 13AB:PO‐T2T:CDBP and 33.0% for DBT‐SADF:PO‐T2T, and to 58.0% for DBT‐SADF:PO‐T2T:CDBP. Therefore, the evidently improved *Φ*_PL_ of DBT‐SADF:PO‐T2T:CDBP is actually ascribed to the better upconversion of triplet excitons with three RISC channels and prove the feasibility of our strategy to improve the exciton utilization of exciplex emitters.

**Table 1:** Summary of photophysical parameters for four exciplex emitters

Emitters	*Φ*_PL_ ^a)^ [%]	*Φ*_P_ ^b)^ [%]	*Φ*_TADF_ ^c)^ [%]	τ_P_ ^d)^ [ns]	τ_TADF_ ^e)^ [µs]	*k*_p_ ^f)^ [×10^7^ s^−1^]	*k*_F_ ^g)^ [×10^7^ s^−1^]	*k*_TADF_ ^h)^ [×10^4^ s^−1^]	*k*_ISC_ ^i)^ [×10^7^ s^−1^]	*k*_RISC_ ^j)^ [×10^5^ s^−1^]
13AB:PO‐T2T	15.0	8.3	6.7	27.8	14.0	3.59	0.30	0.52	3.29	0.05
13AB:PO:T2T:CDBP	40.0	12.0	28.0	14.3	18.6	6.99	0.84	1.71	6.15	0.64
DBT‐SADF:PO‐T2T	38.0	5.0	33.0	23.4	20.5	4.27	0.21	1.69	4.06	1.17
DBT‐SADF:PO‐T2T:CDBP	61.0	3.0	58.0	20.9	16.9	4.78	0.14	3.53	4.64	14.2

^a)^ *Φ*_PL_ is the total photoluminescence fluorescence quantum efficiency value

^b)^ The prompt fluorescence quantum efficiency

^c)^ The delayed fluorescence quantum efficiency

^d)^ Prompt fluorescence lifetime

^e)^ Delayed fluorescence lifetime

^f)^ The rate constants of prompt fluorescence

^g)^ The rate constant of fluorescence decay

^h)^ The rate constants of delayed fluorescence decay

^i)^ The rate constant of intersystem crossing

^j)^ The rate constant of reverse intersystem crossing.

**Figure 3:**
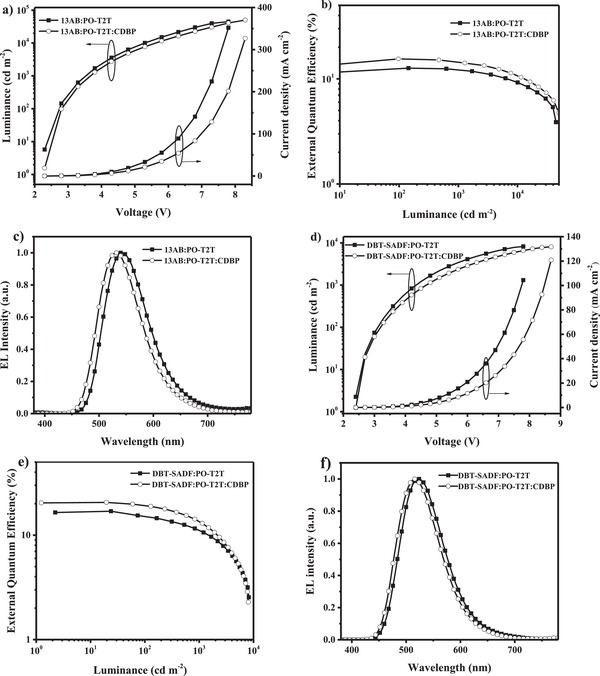
a) Current density–luminance–voltage characteristics; b) EQE–luminance plots; c) EL spectra of the devices based on 13AB:PO‐T2T with a weight ratio of 5:5 and 13AB:PO‐T2T:CDBP with a weight ratio of 1:1:1. d) Current density–luminance–voltage characteristics; e) EQE–luminance plots; f) EL spectra of the OLEDs based on DBT‐SADF:PO‐T2T with a weight ratio of 6:4 and DBT‐SADF:PO‐T2T:CDBP with a weight ratio of 2:5:3.

**Figure 4:**
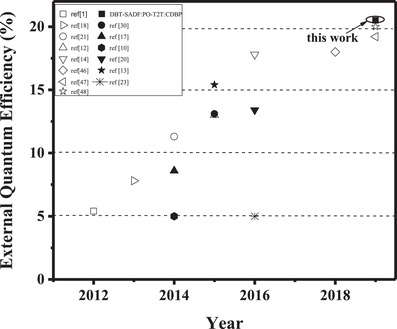
EQE summary of the representative OLEDs based on exciplex emitters.

We finally evaluated the electroluminescence (EL) performance of two tricomponent exciplexes and their contrastive bicomponent exciplexes by using them as the emitters. As the mixing ratios of exciplexes would change their charge balance and affect the device performance,^13^, ^14^ the carrier transporting properties of four exciplexes were carefully investigated. The current density–voltage characteristics of hole‐only and electron‐only devices for four exciplexes with different mixing ratios are shown in Figure S7 in the Supporting Information. And optimal mixing weight ratios with balanced charge transport are 5:5 for 13AB:PO‐T2T, 1:1:1 for 13AB:PO‐T2T:CDBP, 6:4 for DBT‐SADF:PO‐T2T, and 2:5:3 for DBT‐SADF:PO‐T2T:CDBP, respectively. Therefore, the OLEDs using four exciplexes as the emitters were optimized with configurations of ITO/TAPC (35 nm)/13AB (10 nm)/13AB:PO‐T2T (5:5, 30 nm)/PO‐T2T (45 nm)/LiF (1 nm)/Al (100 nm) for 13AB:PO‐T2T, ITO/TAPC (35 nm)/13AB (10 nm)/13AB:PO‐T2T:CDBP (1:1:1, 30 nm)/PO‐T2T (45 nm)/LiF (1 nm)/Al (100 nm) for 13AB:PO‐T2T:CDBP, ITO/TAPC (35 nm)/DBT‐SADF (10 nm)/DBT‐SADF:PO‐T2T (6:4, 30 nm)/PO‐T2T (45 nm)/LiF (1 nm)/Al (100 nm) for DBT‐SADF:PO‐T2T, and ITO/TAPC (35 nm)/CDBP (10 nm)/DBT‐SADF:PO‐T2T:CDBP (2:5:3, 30 nm)/PO‐T2T (45 nm)/LiF (1 nm)/Al (100 nm). In the devices, indium tin oxide (ITO) and Al were served as the anode and the cathode, respectively; TAPC (1,1‐Bis[4‐[*N,N*‐di(*p*‐tolyl)amino]phenyl]‐cyclohexane) and PO‐T2T were used as hole‐transporting layer and electron‐transporting layer, respectively; LiF was acted as electron injection layer; 13AB, DBT‐SADF, and CDBP were used as electron‐blocking layer; exciplexes with the optimal mixing weigh ratios were used as the emitting layer. As listed in **Table** **2**, all four devices exhibit quite low turn‐on voltages around 2.3 and 2.4 V, indicating the superiority of the exciplexes on carriers injection. As shown in **Figure** **3**, both the two tricomponent exciplexes emit same‐shape EL spectra with their corresponding bicomponent exciplexes, except 8 nm blue‐shift. Such blue‐shift is probably caused by the intermolecular interaction between 13AB and CDBP or DBT‐SADF and CDBP, which slightly lowers the HOMO energy levels of 13AB and DBT‐SADF.^47^ With only one RISC channel, the maximum efficiencies of 13AB:PO‐T2T‐based device are 40.4 cd A^−1^, 43.8 lm W^−1^, and 12.4% for current efficiency (CE), power efficiency (PE), and EQE, respectively. The EQE value of 12.4% is far lower than the 20–30% theoretical limit of TADF‐based OLEDs, suggesting significant exciton loss. Correspondingly, 13AB:PO‐T2T:CDBP and DBT‐SADF:PO‐T2T respectively realize maximum CEs of 49.9 and 52.4 cd A^−1^, maximum PEs of 55.9 and 61.0 lm W^−1^, and maximum EQEs of 15.5% and 16.9% in the devices. One additional RISC channel has already improved the device performance of exciplexes evidently, which is consistent with our previous report.^14^ Particularly, with three RISC channels, DBT‐SADF:PO‐T2T:CDBP realizes quite remarkable performance with maximum CE/PE/EQE of 60.0 cd A^−1^, 69.7 lm W^−1^, and 20.5% in the device. From 13AB:PO‐T2T to 13AB:PO‐T2T:CDBP, DBT‐SADF:PO‐T2T to DBT‐SADF:PO‐T2T:CDBP, the maximum EQE values of the devices are evidently improved from 12.4% to 20.5%, suggesting multiple RISC channels can effectively improve the exciton utilization of exciplex emitters. Moreover, to the best of our knowledge, this is the first report with an EQE over 20% for the OLEDs based on exciplex emitters at room temperature (**Figure** **4**; Table S2, Supporting Information),^1^, ^2^, ^4^, ^5^, ^6^, ^7^, ^8^, ^9^, ^10^, ^11^, ^12^, ^13^, ^14^, ^17^, ^18^, ^19^, ^20^, ^21^, ^30^, ^31^, ^46^, ^47^, ^48^ thus our novel strategy of tricomponent exciplexes will provide a valuable approach for further development of exciplex emitters.

**Table 2:** Performance of the OLEDs based on exciplex emitters

Emitter	*V*_on_ ^a)^ [v]	λ_MAX_ [nm]	CE/PE/EQE^b)^ [cd A^−1^/lm W^−1^/%]			CIE^c)^ (x, y)
			Maximum	100 cd m^−2^	1000 cd m^−2^	
mCP:(15%)DBT‐SADF	3.0	500	37.4/35.6/14.0	30.3/26.4/11.4	17.8/13.3/6.7	(0.21, 0.41)
13AB:POT2T	2.3	540	40.4/43.8/12.4	40.1/39.5/12.1	38.1/31.4/11.6	(0.37, 0.57)
13AB:PO‐T2T:CDBP	2.3	532	49.9/55.9/15.5	48.7/46.4/15.1	46.0/38.0/14.1	(0.32, 0.56)
DBT‐SADF:PO‐T2T	2.4	524	52.4/61.0/16.9	44.9/42.8/14.5	32.9/22.9/10.7	(0.29, 0.55)
DBT‐SADF:PO‐T2T:CDBP	2.4	516	60.0/69.7/20.5	54.9/52.3/18.9	41.4/28.9/14.2	(0.26, 0.53)

^a)^ Turn‐on voltage, estimated at 1 cd m^−2^

^b)^ CE, current efficiency; PE, power efficiency; and EQE, external quantum efficiency

^c)^ At 1000 cd m^−2^.

**Figure 5:**
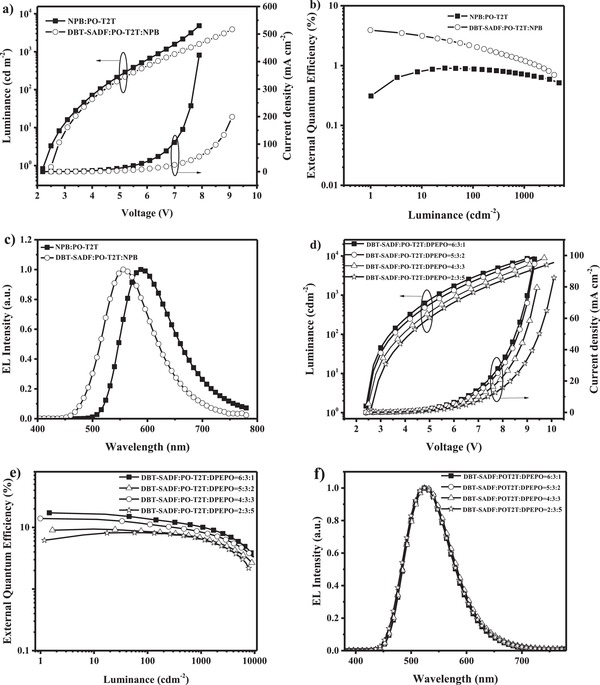
a) Current density–luminance–voltage characteristics; b) EQE–luminance plots; c) EL spectra of the devices based on NPB:PO‐T2T with a weight ratio of 5:5 and DBT‐SADF:PO‐T2T:NPB with a weight ratio of 1:1:1. d) Current density–luminance–voltage characteristics; e) EQE–luminance plots; f) EL spectra of the OLEDs based on DBT‐SADF:PO‐T2T:DPEPO with a weight ratio of 6:3:1, 5:3:2, 4:3:3, and 2:3:5.

To further confirm the proposed multiple reverse intersystem crossing channels in the tricomponent exciplex emitter system, a typical hole‐transport material with (*N*,*N*′‐Di(1‐naphthyl)‐*N*,*N*′‐diphenyl‐(1,1′‐biphenyl)‐4,4′‐diamine (NPB) was chosen to replace the donor CDBP. As NPB has a higher HOMO energy level (−5.3 eV) than DBT‐SADF, the lower‐energy exciplex emitter should be NPB:PO‐T2T. Thus, to well analyze this three‐component exciplex emitter DBT‐SADF:PO‐T2T:NPB, the OLEDs using two exciplexes NPB:PO‐T2T and DBT‐SADF:PO‐T2T:NPB as the emitters were investigated with configurations of ITO/TAPC (35 nm)/NPB (10 nm)/NPB:PO‐T2T (5:5, 30 nm)/PO‐T2T (45 nm)/LiF (1 nm)/Al (100 nm) and ITO/TAPC (35 nm)/DBT‐SADF (10 nm)/DBT‐SADF:PO‐T2T:NPB (1:1:1, 30 nm)/PO‐T2T (45 nm)/LiF (1 nm)/Al (100 nm), respectively. Two devices exhibit quite low turn‐on voltages around 2.3 and 2.5 V. As shown in **Figure** **5**, DBT‐SADF:PO‐T2T:NPB exhibits similar but blue‐shifted EL spectrum compared with NPB:PO‐T2T (respectively peaked at 560 and 588 nm). This behavior is similar with the former two tricomponent exciplex emitters, and probably caused by the intermolecular interaction between NPB and DBT‐SADF, which slightly lowers the HOMO energy level of NPB.^50^ Moreover, the DBT‐SADF:PO‐T2T:NPB‐based device exhibits the maximum efficiencies of 10.9 cd A^−1^, 13.7 lm W^−1^, and 4.1% for CE, PE, and EQE, respectively; while the maximum CE, PE, and EQE of NPB:PO‐T2T‐based device are respectively 2.0 cd A^−1^, 1.84 lm W^−1^, and 0.89%. The maximum efficiencies of the tricomponent device based on DBT‐SADF:POT2T:NPB are nearly five times higher than that of the bicomponent device based on NPB:PO‐T2T. These results further prove both the complete energy transfer from higher‐energy exciplex to lower‐energy exciplex in tricomponent exciplex system and the enhanced excitons utilization of multiple reverse intersystem crossing channels. Meanwhile, we also chose a classical electron‐transporting host material bis[2‐(diphenylphosphino)phenyl]ether oxide (DPEPO) with a wide gap material to replace the donor CDBP molecule, and constructed a tricomponent exciplex system DBT‐SADF:PO‐T2T:DPEPO. As DPEPO has a higher LUMO energy level and HOMO energy level than DBT‐SADF, only exciplex DBT‐SADF:PO‐T2T can be formed in such tricomponent system. The OLEDs based on DBT‐SADF:PO‐T2T:DPEPO were fabricated with configurations of ITO/TAPC (35 nm)/DBT‐SADF (10 nm)/DBT‐SADF:PO‐T2T:DPEPO (30 nm)/PO‐T2T (45 nm)/LiF (1 nm)/Al (100 nm). As shown in Figure 5, with the different weight ratios from 6:3:1 to 5:3:2 and 4:3:3 and 2:3:5, all the devices exhibit nearly consistent EL spectra with the DBT‐SADF:PO‐T2T‐based device. Such results prove that the EL of these devices should be emitted from exciplex DBT‐SADF:PO‐T2T, which is not sensitive to concentration of DBT‐SADF. With a weight ratio of 6:3:1, the device based on DBT‐SADF:PO‐T2T:DPEPO exhibits the maximum efficiencies of 51.7 cd A^−1^, 67.6 lm W^−1^, and 17.3% for CE, PE, and EQE, respectively, which are also nearly consistent with the DBT‐SADF:PO‐T2T‐based device, suggesting the positive impact of the higher‐energy exciplex in tricomponent exciplex systems.

## 3 Conclusion

In conclusion, to further improve the exciton utilization, a novel tricomponent exciplex strategy was proposed to realize exciplex emitters with multiple RISC channels in this work. With a newly designed and synthesized single‐molecule TADF emitter DBT‐SADF, a tricomponent exciplex DBT‐SADF:PO‐T2T:CDBP was developed with three RISC channels accordingly. And DBT‐SADF:PO‐T2T:CDBP successfully realizes much higher *Φ*_PL_ (61.0%) and *k*_RISC_ (14.2 × 10^5^) than other contrasted exciplexes. In the OLED, DBT‐SADF:PO‐T2T:CDBP exhibits a low turn‐on voltage of 2.4 V and high maximum efficiencies of 60.0 cd A^−1^ CE, 69.7 lm W^−1^ PE, and 20.5% EQE. To the best of our knowledge, this is the first report for exciplex emitter–based OLEDs with an over 20% EQE at room temperature. Such high performance demonstrates introducing multiple RISC channels can effectively improve the exciton utilization of exciplex emitters, and our novel strategy of tricomponent exciplexes will provide a valuable approach for further development of exciplex emitters.

## Conflict of Interest

The authors declare no conflict of interest.

## Supporting information

Supplementary

## References

[1] K. Goushi , K. Yoshida , K. Sato , C. Adachi , Nat. Photonics 2012, 6, 253.

[2] T. C. Lin , M. Sarma , Y. T. Chen , S. H. Liu , K. T. Lin , P. Y. Chiang , W. T. Chuang , Y. C. Liu , H. F. Hsu , W. Y. Hung , W. C. Tang , K. T. Wong , P. T. Chou , Nat. Commun. 2018, 9, 3111. 30082702 10.1038/s41467-018-05527-4 PMC6079109

[3] R. Kabe , C. Adachi , Nature 2017, 550, 384. 28967911 10.1038/nature24010

[4] Y.‐S. Park , K.‐H. Kim , J.‐J. Kim , Appl. Phys. Lett. 2013, 102, 153306.

[5] Y. Tao , K. Yuan , T. Chen , P. Xu , H. Li , R. Chen , C. Zheng , L. Zhang , W. Huang , Adv. Mater. 2014, 26, 7931. 25230116 10.1002/adma.201402532

[6] Y. Liu , C. Li , Z. Ren , S. Yan , M. R. Bryce , Nat. Rev. Mater. 2018, 3, 18020.

[7] M. Sarma , K. T. Wong , ACS Appl. Mater. Interfaces 2018, 10, 19279. 29613766 10.1021/acsami.7b18318

[8] M. Mamada , G. Tian , H. Nakanotani , J. Su , C. Adachi , Angew. Chem., Int. Ed. 2018, 57, 12380. 10.1002/anie.201804218 30062688

[9] W. Y. Hung , T. C. Wang , P. Y. Chiang , B. J. Peng , K. T. Wong , ACS Appl. Mater. Interfaces 2017, 9, 7355. 28150488 10.1021/acsami.6b16083

[10] V. Cherpak , P. Stakhira , B. Minaev , G. Baryshnikov , E. Stromylo , I. Helzhynskyy , M. Chapran , D. Volyniuk , Z. Hotra , A. Dabuliene , A. Tomkeviciene , L. Voznyak , J. V. Grazulevicius , ACS Appl. Mater. Interfaces 2015, 7, 1219. 25537396 10.1021/am507050g

[11] D. Chen , G. Xie , X. Cai , M. Liu , Y. Cao , S. J. Su , Adv. Mater. 2016, 28, 239. 26749469 10.1002/adma.201504290

[12] X. K. Liu , Z. Chen , J. Qing , W. J. Zhang , B. Wu , H. L. Tam , F. Zhu , X. H. Zhang , C. S. Lee , Adv. Mater. 2015, 27, 7079. 26436730 10.1002/adma.201502897

[13] X. K. Liu , Z. Chen , C. J. Zheng , C. L. Liu , C. S. Lee , F. Li , X. M. Ou , X. H. Zhang , Adv. Mater. 2015, 27, 2378. 25712786 10.1002/adma.201405062

[14] W. Liu , J.‐X. Chen , C.‐J. Zheng , K. Wang , D.‐Y. Chen , F. Li , Y.‐P. Dong , C.‐S. Lee , X.‐M. Ou , X.‐H. Zhang , Adv. Funct. Mater. 2016, 26, 2002.

[15] M. Colella , P. Pander , D. S. Pereira , A. P. Monkman , ACS Appl. Mater. Interfaces 2018, 10, 40001. 30381946 10.1021/acsami.8b15942

[16] C. Duan , C. Han , R. Du , Y. Wei , H. Xu , Adv. Opt. Mater. 2018, 6, 1800437.

[17] Y.‐S. Park , S. Lee , K.‐H. Kim , S.‐Y. Kim , J.‐H. Lee , J.‐J. Kim , Adv. Funct. Mater. 2013, 23, 4914.

[18] W. Y. Hung , G. C. Fang , Y. C. Chang , T. Y. Kuo , P. T. Chou , S. W. Lin , K. T. Wong , ACS Appl. Mater. Interfaces 2013, 5, 6826. 23848982 10.1021/am402032z

[19] W. Y. Hung , G. C. Fang , S. W. Lin , S. H. Cheng , K. T. Wong , T. Y. Kuo , P. T. Chou , Sci. Rep. 2015, 4, 5161. 10.1038/srep05161 PMC4044637 24895098

[20] S. K. Jeon , K. S. Yook , J. Y. Lee , Nanotechnology 2016, 27, 224001. 27098231 10.1088/0957-4484/27/22/224001

[21] J. Li , H. Nomura , H. Miyazaki , C. Adachi , Chem. Commun. 2014, 50, 6174. 10.1039/c4cc01590h 24781875

[22] B. Zhao , T. Zhang , B. Chu , W. Li , Z. Su , H. Wu , X. Yan , F. Jin , Y. Gao , C. Liu , Sci. Rep. 2015, 5, 10697. 26023882 10.1038/srep10697 PMC4448663

[23] P. Data , P. Pander , M. Okazaki , Y. Takeda , S. Minakata , A. P. Monkman , Angew. Chem., Int. Ed. 2016, 55, 5739. 10.1002/anie.201600113 27060474

[24] V. Jankus , P. Data , D. Graves , C. McGuinness , J. Santos , M. R. Bryce , F. B. Dias , A. P. Monkman , Adv. Funct. Mater. 2014, 24, 6178.

[25] H. G. Kim , K. H. Kim , J. J. Kim , Adv. Mater. 2017, 29, 1702159.

[26] T. Lu , W. Jiang , K. Sun , W. Tian , Y. Sun , Dyes Pigm. 2018, 151, 35.

[27] Y. Seino , H. Sasabe , Y. J. Pu , J. Kido , Adv. Mater. 2014, 26, 1612. 24452829 10.1002/adma.201304253

[28] H. Shin , S. Lee , K. H. Kim , C. K. Moon , S. J. Yoo , J. H. Lee , J. J. Kim , Adv. Mater. 2014, 26, 4730. 24838525 10.1002/adma.201400955

[29] T. Xu , Y. X. Zhang , B. Wang , C. C. Huang , I. Murtaza , H. Meng , L. S. Liao , ACS Appl. Mater. Interfaces 2017, 9, 2701. 28034314 10.1021/acsami.6b13077

[30] K. Goushi , C. Adachi , Appl. Phys. Lett. 2012, 101, 023306.

[31] W. Y. Hung , P. Y. Chiang , S. W. Lin , W. C. Tang , Y. T. Chen , S. H. Liu , P. T. Chou , Y. T. Hung , K. T. Wong , ACS Appl. Mater. Interfaces 2016, 8, 4811. 26820247 10.1021/acsami.5b11895

[32] L. Zhang , C. Cai , K. F. Li , H. L. Tam , K. L. Chan , K. W. Cheah , ACS Appl. Mater. Interfaces 2015, 7, 24983. 26529382 10.1021/acsami.5b05597

[33] W. Liu , C.‐J. Zheng , K. Wang , Z. Chen , D.‐Y. Chen , F. Li , X.‐M. Ou , Y.‐P. Dong , X.‐H. Zhang , ACS Appl. Mater. Interfaces 2015, 7, 18930. 26289611 10.1021/acsami.5b05648

[34] D. Zhang , X. Song , M. Cai , L. Duan , Adv. Mater. 2018, 30, 1705250. 10.1002/adma.201705250 29280207

[35] C. J. Shih , C. C. Lee , T. H. Yeh , S. Biring , K. K. Kesavan , N. R. A. Amin , M. H. Chen , W. C. Tang , S. W. Liu , K. T. Wong , ACS Appl. Mater. Interfaces 2018, 10, 24090. 29943574 10.1021/acsami.8b08281

[36] J. W. Sun , J. Y. Baek , K.‐H. Kim , C.‐K. Moon , J.‐H. Lee , S.‐K. Kwon , Y.‐H. Kim , J.‐J. Kim , Chem. Mater. 2015, 27, 6675.

[37] J. Lee , N. Aizawa , T. Yasuda , Chem. Mater. 2017, 29, 8012. 10.1002/adma.201604856 27859841

[38] D. Zhang , C. Zhao , Y. Zhang , X. Song , P. Wei , M. Cai , L. Duan , ACS Appl. Mater. Interfaces 2017, 9, 4769. 28094502 10.1021/acsami.6b15272

[39] D. Zhang , X. Song , M. Cai , H. Kaji , L. Duan , Adv. Mater. 2018, 30, 1705406. 10.1002/adma.201705406 29315848

[40] H. Uoyama , K. Goushi , K. Shizu , H. Nomura , C. Adachi , Nature 2012, 492, 234. 23235877 10.1038/nature11687

[41] Q. Zhang , B. Li , S. Huang , H. Nomura , H. Tanaka , C. Adachi , Nat. Photonics 2014, 8, 326.

[42] Q. Zhang , H. Kuwabara , W. J. Potscavage Jr. , S. Huang , Y. Hatae , T. Shibata , C. Adachi , J. Am. Chem. Soc. 2014, 136, 18070. 25469624 10.1021/ja510144h

[43] H. Kaji , H. Suzuki , T. Fukushima , K. Shizu , K. Suzuki , S. Kubo , T. Komino , H. Oiwa , F. Suzuki , A. Wakamiya , Y. Murata , C. Adachi , Nat. Commun. 2015, 6, 8476. 26477390 10.1038/ncomms9476 PMC4634127

[44] S. Hirata , Y. Sakai , K. Masui , H. Tanaka , S. Y. Lee , H. Nomura , N. Nakamura , M. Yasumatsu , H. Nakanotani , Q. Zhang , K. Shizu , H. Miyazaki , C. Adachi , Nat. Mater. 2015, 14, 330. 25485987 10.1038/nmat4154

[45] C. Li , C. Duan , C. Han , H. Xu , Adv. Mater. 2018, 30, 1804228. 10.1002/adma.201804228 30306709

[46] M. Guzauskas , D. Volyniuk , A. Tomkeviciene , A. Pidluzhna , A. Lazauskas , J. V. Grazulevicius , J. Mater. Chem. C. 2019, 7, 25.

[47] M. Colella , A. Danos , A. P. Monkman , J. Phys. Chem. Lett. 2019, 10, 793. 30726086 10.1021/acs.jpclett.8b03646 PMC7005938

[48] M. Chapran , P. Pander , M. Vasylieva , G. Wiosna‐Salyga , J. Ulanski , F. B. Dias , P. Data , ACS Appl. Mater. Interfaces. 2019, 11, 13460. 30864778 10.1021/acsami.8b18284

[49] P. L. dos Santos , J. S. Ward , M. R. Bryce , A. P. Monkman , J. Phys. Chem. Lett. 2016, 7, 3341. 27505620 10.1021/acs.jpclett.6b01542

[50] E. Skuodis , A. Tomkeviciene , R. Reghu , L. Peciulyte , K. Ivaniuk , D. Volyniuk , O. Bezvikonnyi , G. Bagdziunas , D. Gudeika , J. V. Grazulevicius , Dyes Pigm. 2017, 139, 795.

