# Supplementary material for: Tricomponent Exciplex Emitter Realizing over 20% External Quantum Efficiency in Organic Light‐Emitting Diode with Multiple Reverse Intersystem Crossing Channels
Source: Adv Sci (Weinh). 2019 May 15;6(14):1801938. doi: 10.1002/advs.201801938 (PMC6661936; doi:10.1002/advs.201801938)
Supplement: Supplementary file 1 — Supplementary [file ADVS-6-1801938-s001.pdf]

Copyright WILEY-VCH Verlag GmbH & Co. KGaA, 69469 Weinheim, Germany, 2019.

ADVANCED SCIENCE
Open Access

Supporting Information

for *Adv. Sci.,* DOI: 10.1002/advs.201801938

Tricomponent Exciplex Emitter Realizing over 20% External Quantum Efficiency in Organic Light-Emitting Diode with Multiple Reverse Intersystem Crossing Channels

Ming Zhang, Wei Liu, Cai-Jun Zheng, Kai Wang, Yi-Zhong Shi, Xing Li, Hui Lin, Si-Lu Tao,* and Xiao-Hong Zhang**

Supporting Information

Tri-Component Exciplex Emitter Realizing Over 20% External Quantum Efficiency in Organic Light-Emitting Diode with Multiple Reverse Intersystem Crossing Channels

Ming Zhang, Wei Liu, Cai-Jun Zheng, Kai Wang, Yi-Zhong Shi, Xing Li, Hui Lin, Si-Lu Tao,* and Xiao-Hong Zhang**

M. Zhang, Prof. C. J. Zheng, Dr. H. Lin, Prof. S. L. Tao
School of Optoelectronic Science and Engineering, University of Electronic Science and Technology of China (UESTC), Chengdu 610054, PR China.
E-mail: zhengcaijun@uestc.edu.cn (C. J. Zheng), silutao@uestc.edu.cn (S. L. Tao)
M. Zhang, W. Liu, Dr. K. Wang, Dr. Y. Z. Shi, X. Li, Prof. X. H. Zhang
Institute of Functional Nano & Soft Materials (FUNSOM), Soochow University, Suzhou 215123, P. R. China.
E-mail: xiaohong_zhang@suda.edu.cn (X. H. Zhang)

Experimental Section

General Information: All reagents were purchased from commercial sources and used as received without further purification. The ^{1}H and ^{13}C NMR spectra were recorded by using a Bruker Advance-400 spectrometer with chemical shifts reported in ppm. MS data were measured via the Finnigan 4021C gas chromatography mass spectrometry instrument. Absorption and PL spectra were measured using a Hitachi UV–vis spectrophotometer U-3010 and a Hitachi fluorescence spectrometer F-4600, respectively. Half-and-half mixed constituting molecules in dichloromethane were coated in quartz tubes and treated at 40 °C under vacuum overnight to form films. Their fluorescence and phosphorescence spectra were measured at 77 K using a Hitachi F-4600 fluorescence spectrometer. The measurement of the phosphorescence spectra was delayed by a chopper with the chopping speed of 40 Hz, corresponding to a delayed time of ≈6.25 ms. The fluorescence quantum yields were measured in N_2 atmosphere with an Edinburgh Instruments FLS920 spectrometer. The temperature-dependent transient PL decay characterizations were conducted by the Collaborative Innovation Center of Suzhou Nano Science & Technology. Cyclic voltammetry was performed on a CHI660E electrochemical analyzer with 0.1 MBu_4NPF_6 as a supporting electrolyte, a saturated calomel electrode (SCE) as the reference electrode, a Pt disk as the working electrode, and a scan rate of 10 mV s^{-1}. The oxidation potential of SCE relative to the

vacuum level is calibrated to be 4.60 and 4.52 V in acetonitrile (ACE) and dimethylformamide (DMF). The oxidation potentials of 13AB, CDBP, DBT-SADF were conducted in ACE, and the reduction potentials of PO-T2T was conducted in DMF. All film samples used for the photophysical measurements were fabricated by vacuum deposition.

OLEDs Fabrication: ITO-coated glasses with a sheet resistance of 15 Ω square^{-1} were first cleaned with isopropyl alcohol and deionized water, then dried in an oven at 120 °C, treated with UV-ozone, and finally transferred to a deposition system with a base pressure of about 4 × 10^{-4} Pa. Organic materials were deposited at a rate of 1–2 Å s^{-1} and the rates were 0.1 and 10 Å s^{-1} for LiF and Al, respectively. The emitting layer contained three compounds was evaporated from three different evaporators with independent monitoring. EL luminescence, spectra, and CIE color coordinates were measured with a Spectrascan PR650 photometer and the current–voltage characteristics were measured using a Keithley 2400 SourceMeter under ambient atmosphere. EQE was calculated from the current density, luminance, and EL spectrum, assuming a Lambertian distribution.

Materials and Synthesis

All commercially available reagents and chemicals were used without further purification.

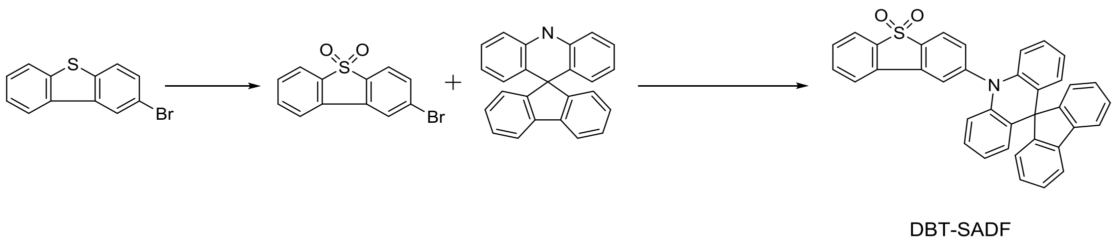

Scheme S1. Molecule structure and Synthetic route of DBT-SADF.

2-(10H-spiro[acridine-9,9'-fluoren]-10-yl)dibenzo[b,d]thiophene 5,5-dioxide. The 2-Bromodibenzothiophene (1.315 g, 5 mmol) was oxidized by dissolution in 80 mL of acetic acid, addition of 40 mL of 30% H_2O_2, and warming slowly first for 1.5 h at 45 °C and then for 1 h at 75 °C. The cooled reaction was diluted with 400 mL of water, and the resulting solid was removed by filtration, washed with water, and dried, followed by crude chromatography in Petroleum ether, yielding 1.196 g of white solid (91%). MS (EI) m/z: [M]+: calcd for C12H7BrSO_2 295.1527; found, 295.1562. Then, toluene (20 ml) and tri-tert-butyl phosphine solution 10% in pentane (0.3 mL, 0.13 mmol) were added to a mixture of 2-bromodibenzo[b,d]thiophene 5,5-dioxide (295.15

mg, 1 mmol), 10H-spiro[acridine-9,9'-fluorene] (364.56mg,1.1 mmol), palladium acetate (6.77 mg, 0.05 mmol), and sodium tert-butoxide (288.31 mg, 3 mmol). With stirring, the suspension was heated at 90°C for 24 h under nitrogen atmosphere. When cooled to room temperature, the mixture was extracted with dichloromethane and dried over Na_2SO_4. After the solvent had been removed, the residue was purified by column chromatography on silica gel using dichloromethane as the eluent to give a white solid, with an 82.1 % yield (448 mg). ^{1}H NMR (400 MHz, $CDCl_3$) δ 8.19 (d, *J* = 7.9 Hz, 1H), 7.95 – 7.90 (m, 2H), 7.83 (dd, *J* = 12.5, 7.7 Hz, 3H), 7.70 (t, *J* = 6.8 Hz, 2H), 7.62 (t, *J* = 7.5 Hz, 1H), 7.40 (s, 2H), 7.38 (s, 1H), 7.26 (dd, *J* = 14.3, 7.1 Hz, 3H), 6.95 (t, *J* = 7.7 Hz, 2H), 6.62 (t, *J* = 7.5 Hz, 2H), 6.44 (d, *J* = 7.8 Hz, 2H), 6.37 (d, *J* = 8.4 Hz, 2H). ^{13}C NMR (100 MHz, $CDCl_3$) δ 155.97, 146.52, 139.97, 138.79, 137.61, 136.95, 134.80, 133.69, 133.28, 130.64, 130.23, 127.96, 127.81, 127.28, 127.00, 125.20, 124.66, 124.63, 124.53, 122.03, 121.65, 120.91, 119.55, 113.92, 56.14. HRMS (EI) m/z: [M]+: calcd. for $C_{37}H_{23}NO_2S$:545.3049; found: 545.3094.

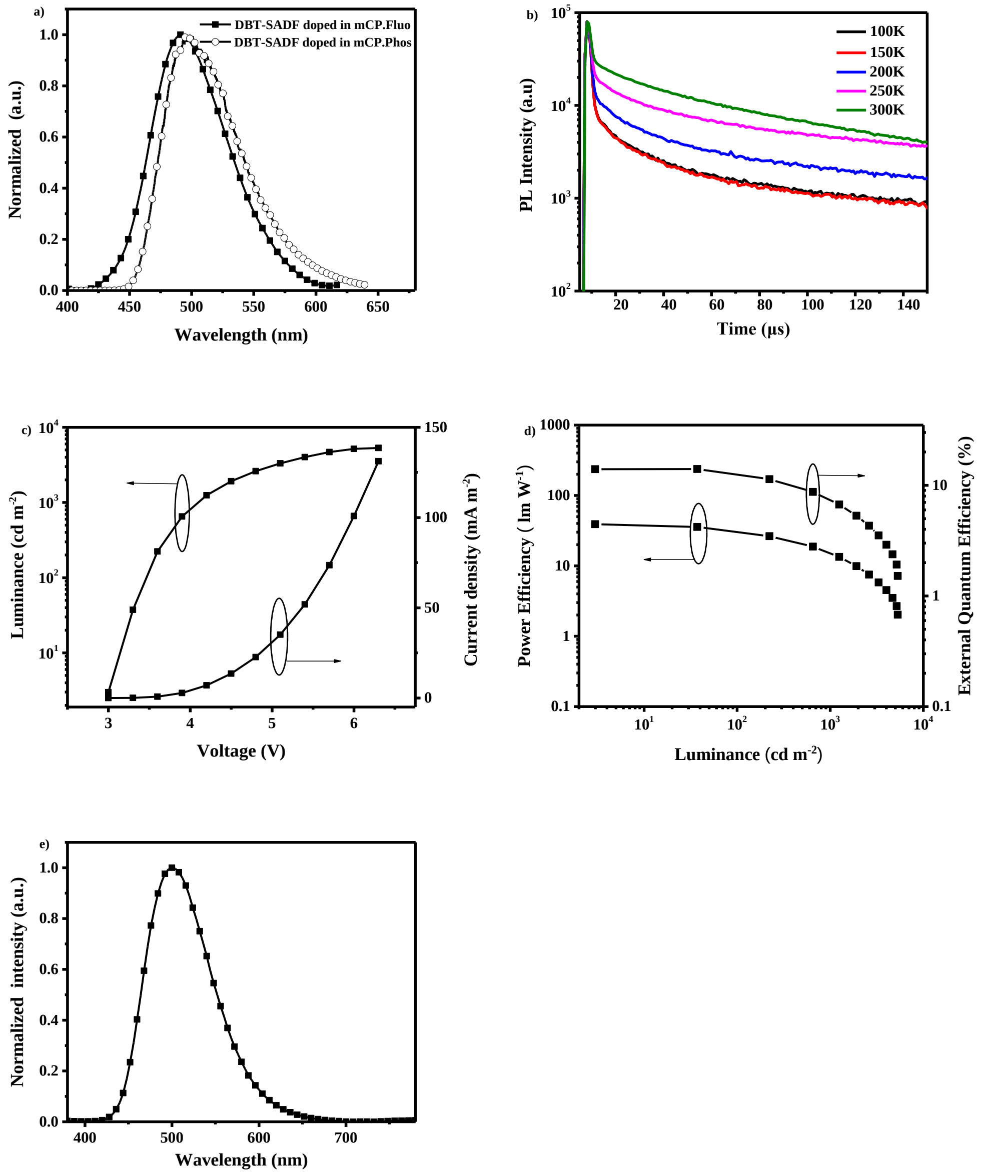

Figure S1. a) Fluorescence and phosphorescence spectra of DBT-SADF in mCP film at 77K; b) transient PL decay of a DBT-SADF doped mCP film at 498 nm with 300 nm excitation in vacuum; c) Current density–luminance-voltage characteristics; d) EQE–PE-luminance plots; e) EL spectra of the devices based on DBT-SADF doped in mCP. The optimized structure is ITO/TAPC (35 nm)/TCTA (10 nm)/mCP:15 wt% DBT-SADF (20 nm)/TmPyPb (40 nm)/LiF (1 nm)/Al (100 nm).

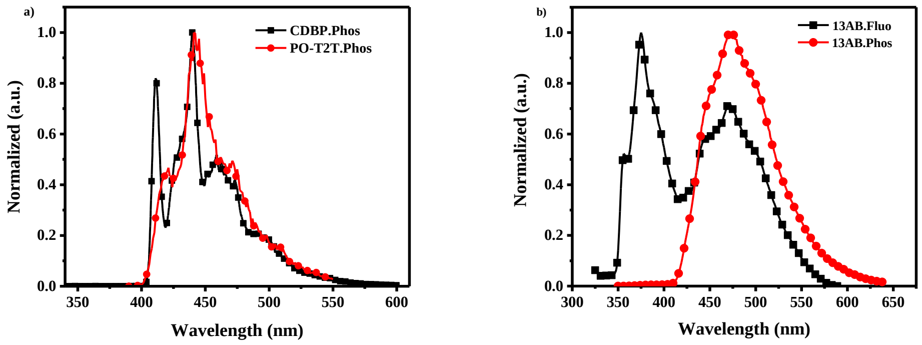

Figure S2. a) the phosphorescence spectra of CDBP, PO-T2T in 2Me-THF at 77 K; b) the Fluorescence and phosphorescence spectra of 13AB in film state at 77 K.

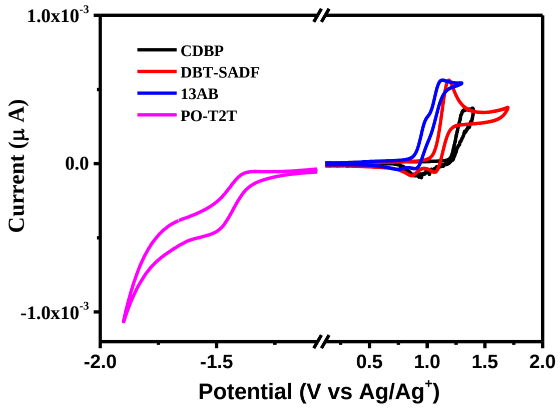

Figure S3. a) Oxidation curves of CDBP, 13AB, DBT-SADF in ACE and reduction curve of PO-T2T in DMF. The HOMO level of CDBP, 13AB, DBT-SADF/LUMO level of PO-T2T are calculated with equation: HOMO = − $e(E_{ox} + 4.60)$/LUMO = − $e(E_{red} + 4.52)$. E_{ox}, E_{red} are the onsets of oxidation and reduction curves.

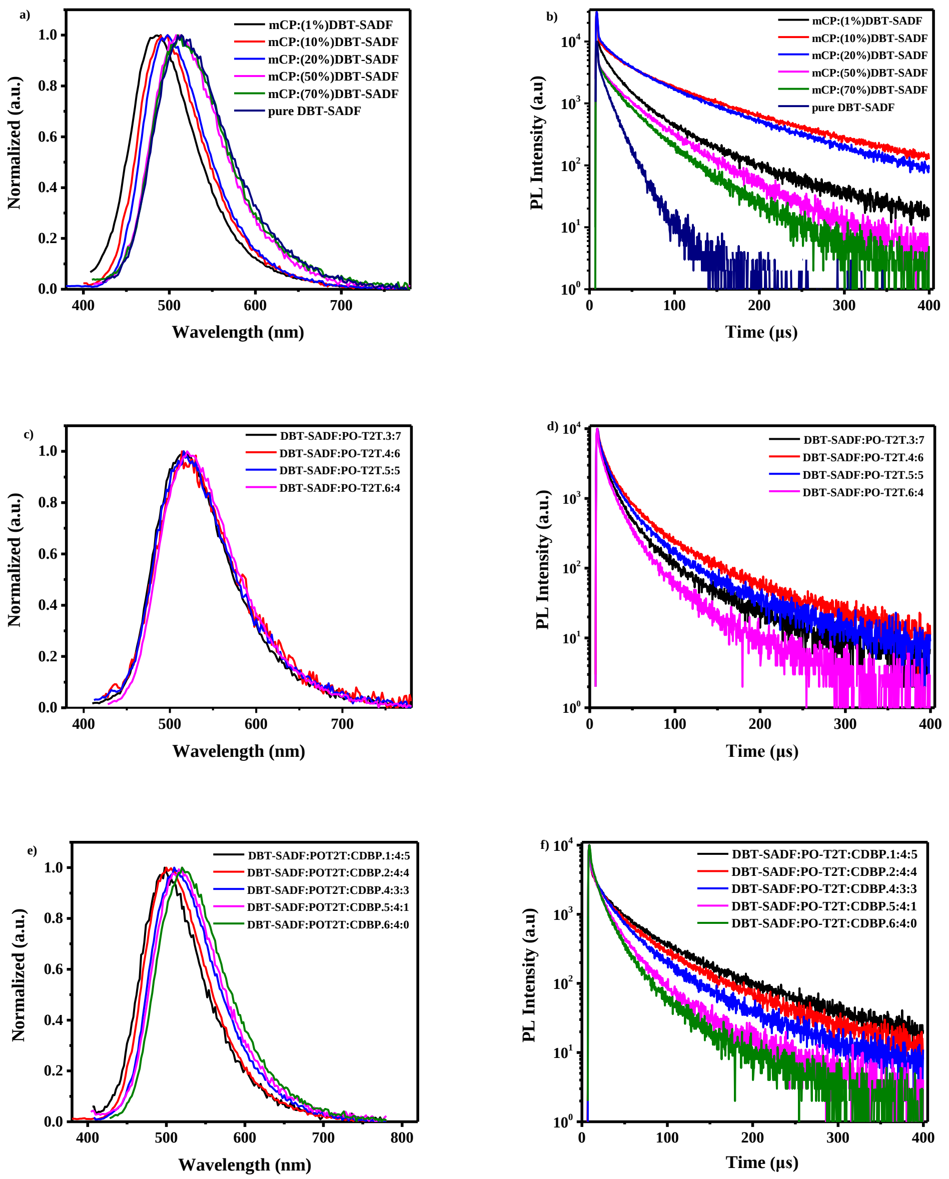

Figure S4. Fluorescence and transient fluorescence decays (measured with 300 nm excitation in vacuum) of the mixed films: a) and b) DBT-SADF doped in mCP, 1%, 10%, 20%, 50%, 70%, 100% with the peaks at 490 nm, 498 nm, 498 nm, 508 nm, 510 nm, 514 nm; c) and d) DBT-SADF:PO-T2T with a ratio weight at 3:7, 4:6, 5:5, 6:4 with the peaks at 516 nm, 516 nm, 516 nm, 518 nm; e) and f) DBT-SADF:PO-T2T:CDBP with a ratio weight at 1:4:5, 2:4:4, 4:3:3, 5:4:1, 6:4:0 with the peaks at 500 nm, 500 nm, 510 nm, 520 nm, 524 nm.

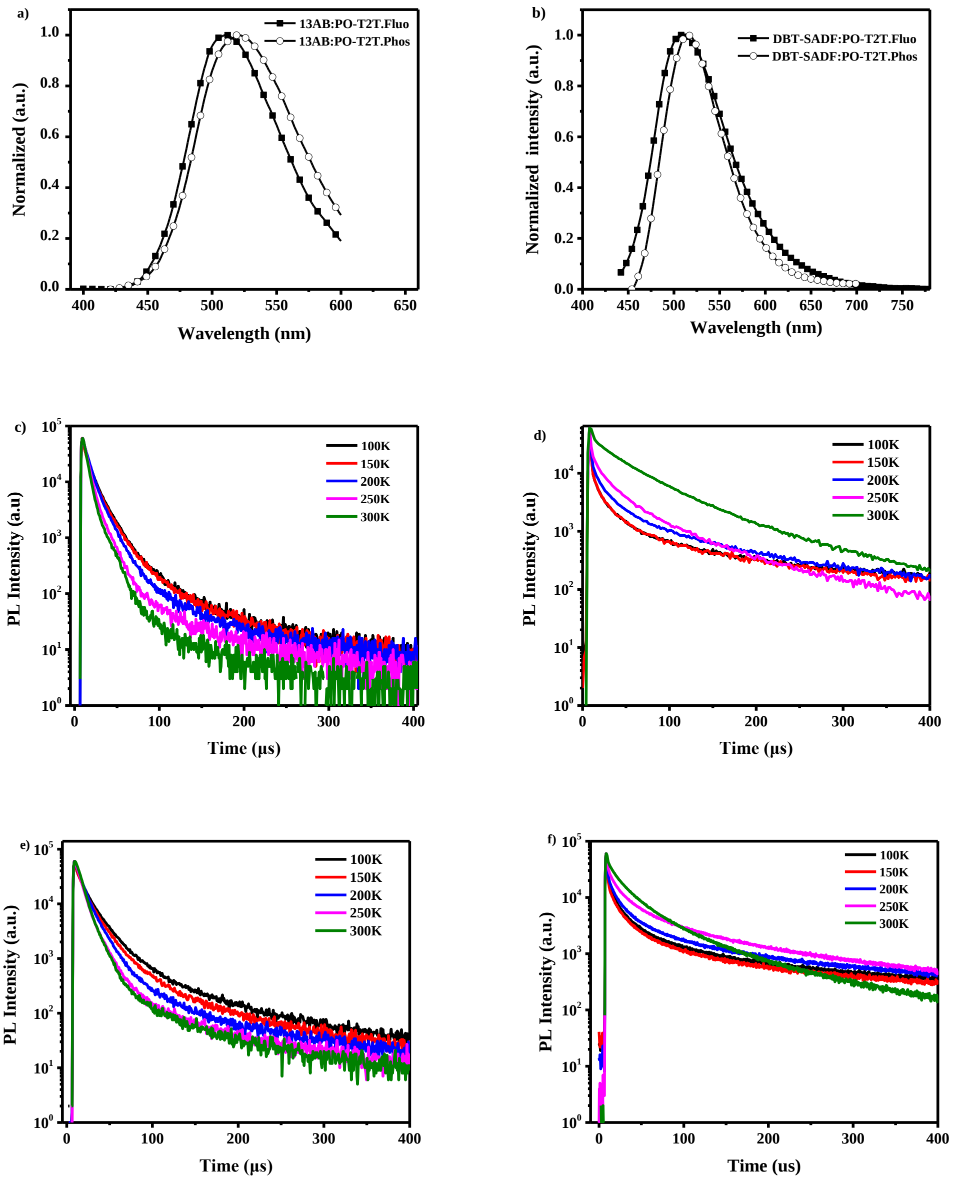

Figure S5. Fluorescence and phosphorescence spectra of mixed films at 77 K: a) 13AB:PO-T2T; b) DBT-SADF:PO-T2T. Temperature-dependent transient fluorescence decays of the mixed films: c) 13AB:PO-T2T at 534 nm with 300 nm excitation in vacuum; d) DBT-SADF:PO-T2T at 514 nm with 300 nm excitation in vacuum; e) 13AB:PO-T2T:CDBP at 516 nm with 300 nm excitation in vacuum; f) DBT-SADF:PO-T2T:CDBP at 510 nm with 300 nm excitation in vacuum.

Table S1. Summary of the key physical properties of these exciplexes emitters and their constituting materials.

Compound	$\lambda_{flou.}$ [a] [nm]	S_1 [b] [eV]	T_1 [c] [eV]	ΔE_{ST} [d] [eV]	HOMO [e] [eV]	LUMO [f] [eV]
13AB	381	3.57	3.01	0.56	-5.60	---
CDBP	367	---	3.02	---	-5.83	---
PO-T2T	327	---	2.95	---	---	-3.22
DBT-SADF	497	2.520	2.490	0.03	-5.65	---
CDBP:PO-T2T	478	2.691	2.668	0.023	---	---
13AB:PO-T2T	522	2.428	2.381	0.047	---	---
DBT-SADF:PO-T2T	516	2.442	2.410	0.032	---	---

[a] Determined from the emission peak of their film at room temperature; [b] Estimated from the peak of fluorescence spectrum at 77K; [c] Estimated from the peak of phosphorescence spectrum at 77K; [d] $\Delta E_{ST} = S_1 - T_1$; [e] Determined from the onset of oxidation potential with respect to that of ferrocene in ACE solution; [f] Determined from the onset of the reduction curve with respect to that of ferrocene in DMF solution.

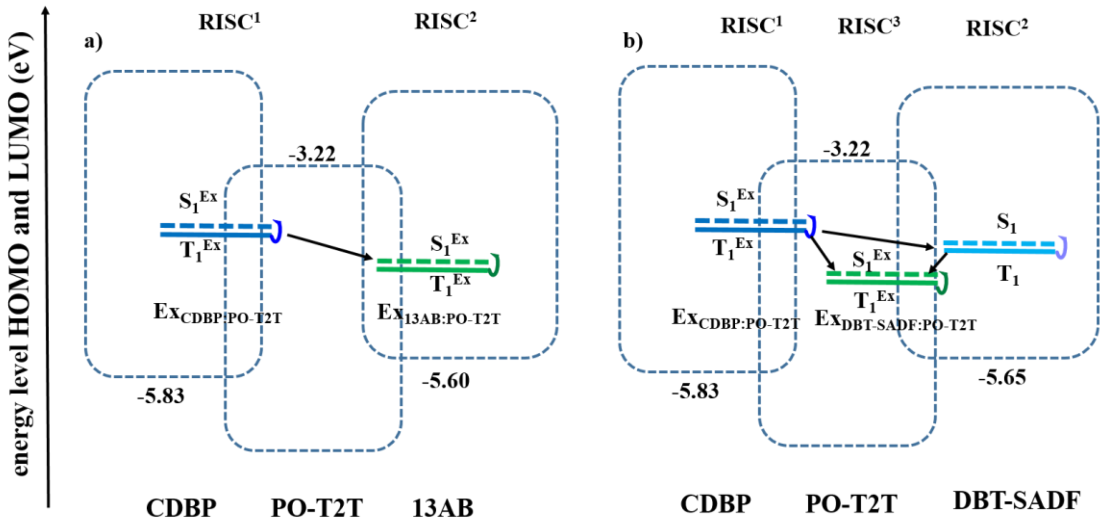

Figure S6. Energy transfer diagrams of the novel tri-component exciplex emitters. a) 13AB:PO-T2T:CDBP with two RISC channels; b) DBT-SADF:PO-T2T:CDBP exciplex with three RISC channels.

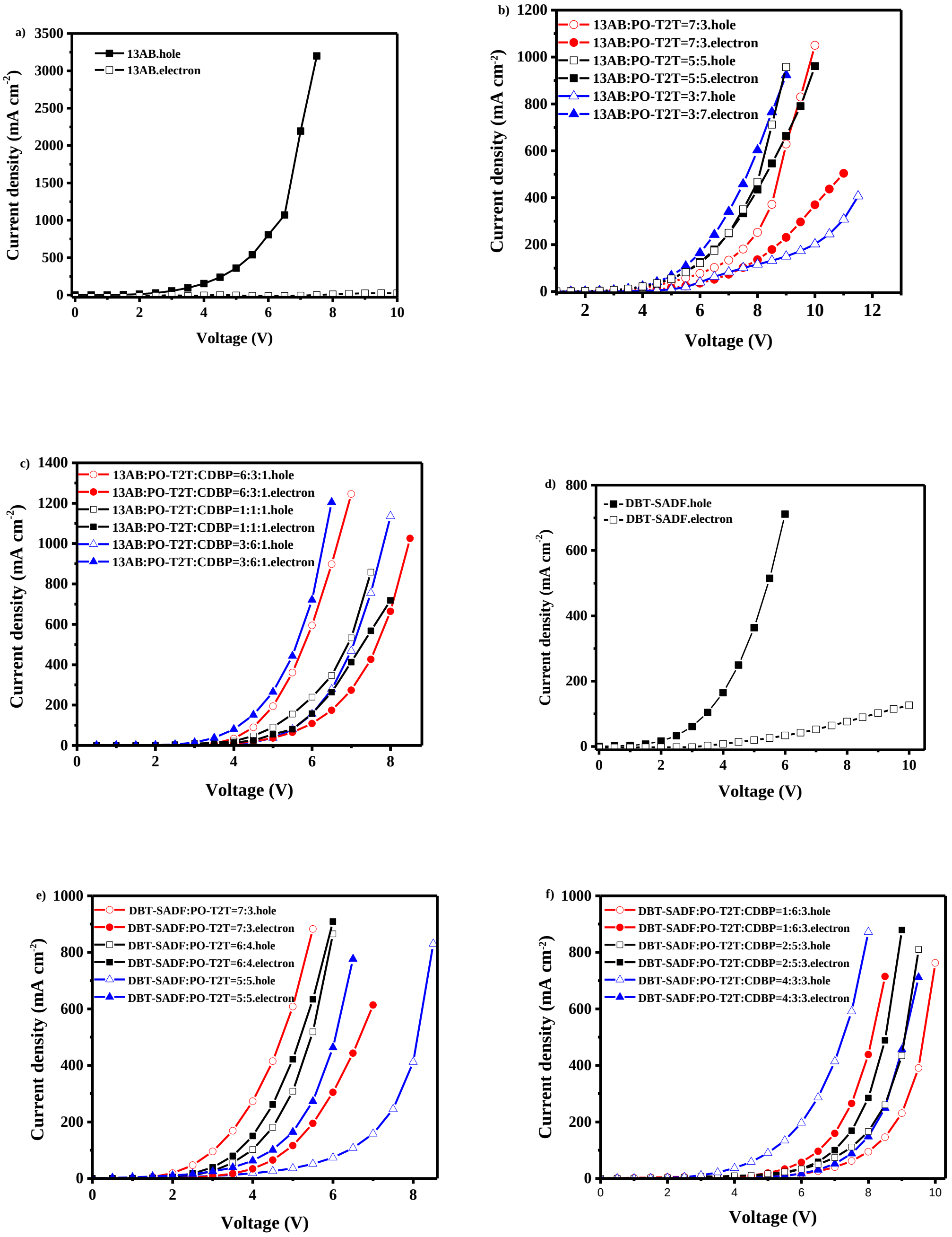

Figure S7. Current density–voltage characteristics of a) 13AB; b) 13AB:PO-T2T; c) 13AB:PO-T2T:CDBP; d) DBT-SADF; e) DBT-SADF:PO-T2T; f) DBT-SADF:PO-T2T:CDBP. Hole-only devices: ITO/MoO_3 (12 nm)/exciplex emitter (50 nm)/MoO_3 (12 nm)/Al (100 nm); Electron-only devices: ITO/Al (40 nm)/LiF (1 nm)/exciplex emitter (50 nm)/LiF (1 nm)/Al (100 nm).

Table S2. Summary of high-efficiency TADF OLEDs based on exciplex emitters and single-molecule TADF emitters.

		Emitters	V_{on} [a)] [V]	EQE_{max} [b)] [%]	EQE[%] at 100 cd m^{-2}	EQE[%] at 1000 cd m^{-2}	CIE [c)] (x, y)
Exciplexes	This work	DBT-SADF:PO-T2T:CDBP	2.4	20.5	18.9	14.3	(0.26, 0.53)
	Ref. [14]	MAC:PO-T2T	2.4	17.8	16.1	12.3	(0.31, 0.55)
	Ref. [13]	TAPC:DPTPCz	2.7	15.4	12.0	8.3	(0.27, 0.52)
	Ref. [30]	TCTA:Tm3PyBPZ	2.4	13.1	11.8	—	—
	Ref. [19]	mCP:HAP-3MF	4.0	11.3	10.5	8.0[d)]	—
	Ref. [28]	m-MTDATA:PPT	—	10.0	7.6	4.0[d)]	—
	Ref. [17]	mCP:PO-T2T	2.0	8.0	4.1	3.5[d)]	(0.17, 0.23)
	Ref. [9]	TCTA:3P-T2T	2.0	7.8	7.8	7.7	(0.40, 0.55)
	Ref. [1]	m-MTDATA:3TPYMB	—	5.4	—	—	—
Single TADF molecule	Ref. [36]	DMAC-DPS	>4.0	19.5	18.0[d)]	16.0[d)]	(0.16, 0.20)
	Ref. [33]	DPTDDA	3.0	22.3	21.0[d)]	10.6[d)]	(0.14, 0.19)
	Ref. [37]	b1	3.0	12.5	9.0[d)]	2.5[d)]	(0.61, 0.39)
	Ref. [38]	DACT-II	2.6	29.6	26.5[d)]	22.8[d)]	—
	Ref. [35]	4CzIPN	—	19.3	—	—	—
	Ref. [31]	CPC	3.2	21.2	16.1	9.7	(0.19, 0.43)
	Ref. [34]	MXAc-BF	3.0	16.2	15.7	12.0	(0.17, 0.29)
	Ref. [45]	ptBCzPO2TPTZ	2.9	28.9	26.1	16.4	(0.18, 0.42)

Turn-on voltage, estimated at the brightness of 1 cd m^{-2}; b) Maximum external quantum efficiency; c) Voltage at which emission became detectable; d) Estimated from the graphs in the references. All cited references were placed in the text.

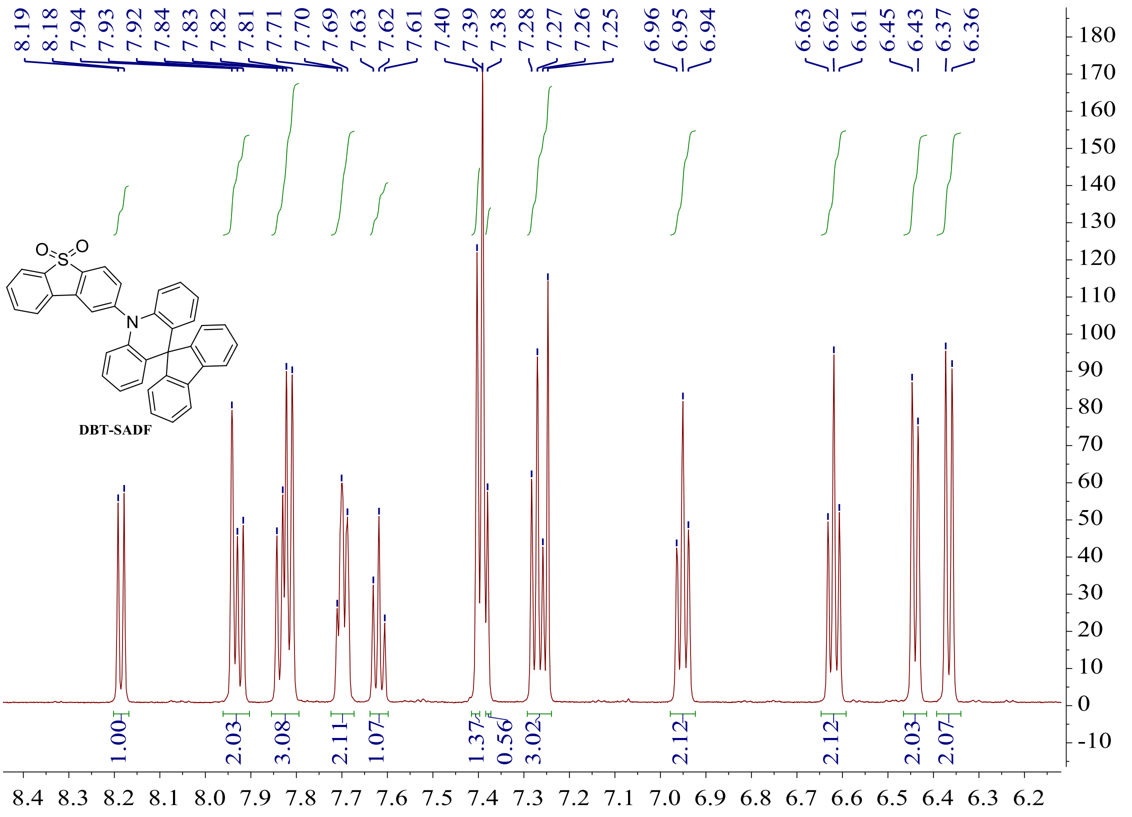

Figure S8. ^{1}H NMR spectrum of DBT-SADF.

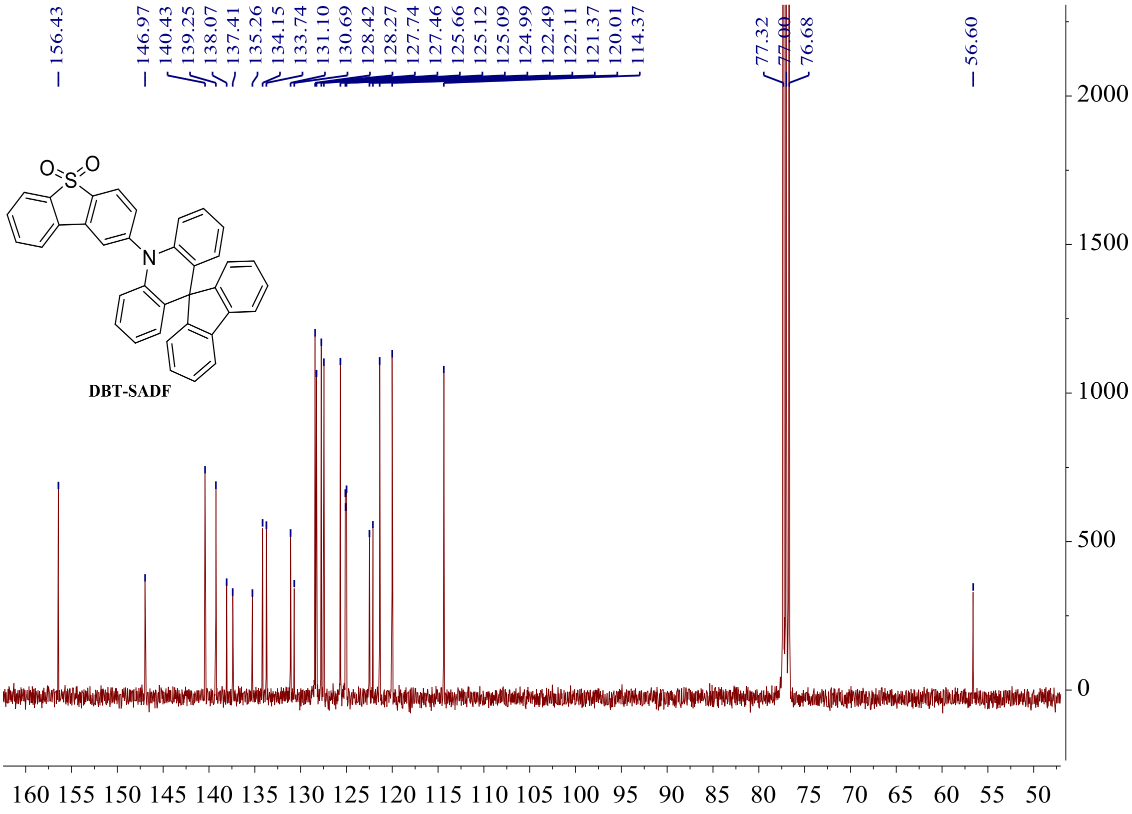

Figure S9. ^{13}C NMR spectrum of DBT-SADF.
